# Climate Change and Social Campaigns

**DOI:** 10.25122/jml-2020-0173

**Published:** 2020

**Authors:** Raluca Raducu, Cristina Soare, Cristina-Mihaela Chichirez, Monica Roxana Purcarea

**Affiliations:** 1. Department of Marketing and Medical Technology, “Carol Davila” University of Medicine and Pharmacy, Bucharest, Romania; 2. Department of Nephrology, “Carol Davila” Clinical Nephrology Hospital, Bucharest, Romania

**Keywords:** Civil society, climate activism, climate change, demonstrations, social campaigns

## Abstract

The impact of climate change on humanity and nature is increasingly evident. The atmosphere and oceans have warmed, leading to rising sea levels, a sharp drop in Arctic sea ice, floods, heatwaves, and fires. Calls to action are getting stronger. Concerns about climate change have become a full social movement, stimulating climate activism from the bottom up to the world, especially among young people. Campaigns are initiated by governments and international organizations, scientists and scientific institutions, organizations, groups, and people in civil society, public intellectuals and political, religious leaders, people of culture and entertainment. These campaigns generally aim to inform, raise awareness and shape public understanding about the science, problems, and policy of climate change, with the hope that, first of all, people will change their attitudes and behavior, and secondly, will mobilize to put pressure on policymakers for effective climate policies.

Climate change is one of the greatest threats facing humanity at the moment. The causes are often intangible, and the impact is broad and difficult to predict for certain areas and periods of time.

As global temperatures rise, accelerated melting of glaciers, rising sea levels, and record storms, as well as severe floods, dry and fire, the warnings from the scientific community are becoming more and more urgent.

There is an almost global consensus among the scientific community that highlights a causal relationship between human activities and climate change, with compelling evidence that climate change results from the combination of natural variability and human influences, especially greenhouse gases, resulting from the use of fossil fuels and changes in the utilization of the land [1].

Climate change not only causes an increase in the frequency and severity of extreme weather events, such as floods, hurricanes, fires, dry, heat waves, but also generates longer-lasting changes, such as sea-level rise, land desertification, entire areas becoming uninhabitable [2]. These changes affect food security, water availability, a number of health conditions, home security, agricultural productivity, and natural ecosystems [3].

The causes and the impacts of climate change are unequal, being produced disproportionately by developed countries, and the effects are most felt by developing countries. Children and future generations will bear the worst consequences [4].

Climate change has been the subject of increased international attention, especially after taking the effect of the Kyoto Protocol, which is the first step towards reducing global human influence on the climate system.

The Kyoto Protocol is an international treaty extending the 1992 United Nations Framework Convention on Climate Change (UNFCCC), adopted on December 11, 1997, in Kyoto, Japan, and took effect on February 16, 2005.

The Protocol implemented the UNFCCC's objective of slowing the onset of global warming by reducing greenhouse gas concentrations in the atmosphere to a level that would prevent dangerous anthropogenic interference with the climatic system [5]. The UNFCCC aims to stabilize greenhouse gas concentrations in the atmosphere at a level where ecosystems can naturally adapt to climate change. Food production is not threatened, and economic development can continue in a sustainable way [6].

In 2010, the UNFCCC parties agreed that future global warming should be limited to less than 2°C related to pre-industrial levels [7]. According to the 2015 Paris Agreement, this fact was confirmed. With the 1.5°C Special Report on Global Warming, the International Climate Change Group highlighted the benefits of keeping global warming below this level, suggesting a global collective effort that can be guided by the 2015 United Nations Sustainable Development Goals.

A report published in 2019, before the UN Climate Action Summit, said that the implementation of all commitments made by all international coalitions would be sufficient to limit temperature rise to 2°C, but not to 1.5°C [8].

The limiting of global warming to 1.5°C will require a rapid and profound change in attitudes, rules, incentives, and policies. Some of the key puzzles on climate change and energy transition are in the area of social sciences. However, the social sciences field receives the lowest funding for climate research. It has been estimated that only 0.12% of all climate research funds are spent on the social science of climate change mitigation [9].

Concerns about climate change have become a full social movement, stimulating climate activism from the bottom up worldwide, especially among young people. Since the Rio Earth Summit in 1992, young people have participated in international negotiations that have looked at various environmental and sustainable development issues.

With the formation of the European Youth Forum in 1996 and the US Youth Organization SustainUS in 2001, youth organizations began to send delegations for active participation in various global negotiations, mainly through the United Nations. 

After the United Nations Convention on Climate Change in Montreal, Canada, from November 28 to December 9, 2005, youth delegations from member countries, which participated in international climate negotiations, were formed for the first time in an international organism called Youth Climate Movement [10].

After that, around the world, youth organizations formed climate coalitions that joined the Youth Climate Movement. In September 2006, 48 youth organizations from Canada met to discuss climate change, and they formed the Canadian Youth Climate Coalition (CYCC) [11]. The coalition acts as a pressure group to encourage politicians to take action on climate change. Between 2007 and 2011, the Canadian Youth Climate organized a youth delegation at the United Nations Climate Change Conferences to convey the voice of the Canadian climate change movement. In 2009 and 2011, CYCC organized the Canadian Power Shift conference. In 2010, it offered young training camps for climate trainers, and it organized a campaign to encourage and empower young people across the country to meet with their members from the parliament to ensure that the government knows that young people are involved and are taking a position for a better and sustainable future [12].

In November 2006, the Australian Youth Climate Coalition (AYCC) was formed with 27 youth organizations. Its goal is to educate, inspire, and mobilize an entire generation to fight for climate justice and a clean energy future [13]. Every year since the establishment of the Australian climate change coalition, the organization has sent its delegations to all United Nations conferences to advocate for young people.

In September 2009 and 2011, the AYCC organized Youth Decide with World Vision Australia. It was Australia's first national climate vote giving young people the opportunity to vote on the renewable energy targets they wanted to set with the federal government.

During the 2010 election campaign, the AYCC mobilized hundreds of young people to get climate change back on the political agenda. This included automated phone calls to politicians and the presentation of scoreboards assessing the climate policies of the three main political parties [14].

Through the Repower Port Augusta campaign from 2012, when 100 young people traveled 328 km in 15 days from Port Augusta to Adelaide, they promoted investments in the first thermal solar power plant, the event gaining significant political attention and media.

AYCC has over 100,000 members now, and it has become one of the most effective social change organizations in Australia [15].

The UK Youth Climate Coalition (UKYCC) is a non-profit youth organization, led entirely by volunteers, formed in 2008 by bringing together a number of youth organizations and a coalition of non-governmental organizations. The organization's mission is to mobilize and empower young people to take positive action for global climate justice.

The UKYCC vision is a right, sustainable world where current and future generations enjoy and protect a healthy environment [16].

The organization is divided into several working groups, with activities on system change, community issues, and activism, climate issues.

The China Youth Climate Action Network (CYCAN) was formed in August 2007 from 7 youth organizations, with the mission to coordinate and provide services to interested organizations in promoting youth participation in climate change actions. It also carries out extensive advertising activity to raise public awareness of climate change and the transition to sustainable energy. More than 300 colleges and universities participated in activities organized and sponsored by CYCAN.

The campaign against climate change (CCC or CaCC) is a UK-based pressure group that aims to raise awareness of anthropogenic climate change by mobilizing mass demonstrations. Founded in 2001, in response to President Bush's rejection of the Kyoto Protocol, the organization stood out by a presence at the demonstrations from October to December 2005. An estimated 10,000 people attended the London rally in December 2005.

Simultaneously, demonstrations have been scheduled in more than 30 countries around the world to coincide with the crucial talks in Montreal on preliminary agreements for a post-Kyoto treaty to take effect after 2012. The protests in December 2006 had international recognition, with the London protest attracting more than 10,000 participants [17].

The campaign against climate change was heavily involved in demonstrations against the closure of the Vestas wind turbine plant on the Isle of Wight and the occupation of the factory by workers, and it was involved in the mobilizations for the demonstrations that were marked the United Nations climate discussions in Copenhagen in December 2009. 

The People's Climate Movement (PCM) is a coalition of civil organizations, including environmental and religious organizations, unions, and social justice groups in the US, that advocate for political and social change to address the effects of climate change. Since 2014, it has been holding marches in the US to raise awareness and call for action on climate issues. In 2018, PCM led the Rise for Climate, Jobs and Justice March in San Francisco with over 30,000 participants, while organizing several events in the US [18].

The People's Climate March was initiated by Bill McKibben, author, and environmentalist, who strongly criticized the inaction of world leaders in tackling the problem [19]. Most of his criticism was in the form of articles that spread worldwide through the media and the Internet. His campaign was particularly successful at the 2014 UN Climate Summit in New York when his environmental organization '350.org.' along with 1,500 other international organizations marched in New York as a protest against the negligence of world leaders [20]. This initiative can be seen as a core movement triggered by a specific action and campaign, which continues to engage a multitude of people around the world, giving them guidance on how to organize their own campaigns using soft power tools. 

Worldwide, it is estimated that over 600,000 people participated in these demonstrations. The 2017 march was a protest against the environmental policies of US President Donald Trump and his administration. The protests took place at the end of his first 100 days as president, attended by 200,000 people.

Online communication campaigns emerged in the early 2000s, playing a key role in shaping what people think, feel, and do about climate change while influencing decision-makers at a local, national and international level. As more and more people around the world have access to the Internet, online and social media are becoming significant contexts that include news, debate, action, and social contributions related to climate change. The campaigns are initiated by governments and international organizations, scientists and scientific institutions, organizations, groups and people in the civil society, public intellectuals and political, religious leaders, people of culture and entertainment.

These campaigns generally aim to inform, raise awareness and shape public understanding about the science, problems and policy of climate change, with the hope that, first of all, people will change their attitudes and their behavior, and secondly, they will mobilize to put pressure on decision-makers.

Independent activists have played a significant role in the fight against climate change. Demonstrations have become increasingly popular among young people, who are aware of the consequences of global warming and how they will affect their future. What began as a protest by a 16-year-old Swedish girl, Greta Thunberg, became a global campaign in 2018, joined by more than 20,000 students. Greta Thunberg was invited to speak on behalf of young people at various international courts [21]. As part of the global movement on school strikes, Greenhouse PR has mobilized support for the UK's Student Climate Network and helped increase participation in the largest climate strike in history. Thus, on September 20, 2019, more than 350,000 people took to the streets in the UK, creating unprecedented interest in climate protests, showing huge public support for urgent climate action that exists around the world.

Lately, public figures have focused on raising awareness of climate change through effective campaigns using advances in technology. Thus, online platforms have been used both to raise awareness and mobilize volunteers and movements to address the adverse effects of climate change in order to influence political decision-making [22,23].

Climate change is a global problem that requires a collective response. The discussion at the local level is an essential element in propelling this collective response, often through the mechanism of social campaigns.

Social and civic engagement, important for rapid climate action and living in a climate-changing world, includes volunteering and joining community groups and engaging in civic citizenship.

In recent years, social marketing efforts have focused very much

on the area of behavioral problems related to environmental protection and the successful implementation of the principles and techniques of social marketing could be beneficial.

## Conflict of Interest

The authors declare that there is no conflict of interest.
